# Evaluation of a mobile app for dark adaptation measurement in individuals with age-related macular degeneration

**DOI:** 10.1038/s41598-023-48898-5

**Published:** 2023-12-14

**Authors:** Shrinivas Pundlik, Prerana Shivshanker, Archana Nigalye, Gang Luo, Deeba Husain

**Affiliations:** 1 Schepens Eye Research Institute of Mass Eye and Ear, 20 Staniford Street, Boston, MA 02114 USA; 2 Retina Service, Massachusetts Eye and Ear Infirmary, Boston, USA; 3 Department of Ophthalmology, Harvard Medical School, Boston, USA

**Keywords:** Macular degeneration, Translational research, Diagnostic markers, Prognostic markers

## Abstract

We present clinical evaluation of a mobile app for dark adaptation (DA) measurement in age-related macular degeneration (AMD) patients and in older adults (age > 50 years) without AMD or other retinal disorders (NV). The outcome measures were the area under dark adaptation curve (AUDAC) and the time for visual sensitivity to recover by 3 log units (T_R_). Larger AUDAC and T_R_ values indicated worse DA response. The association of AUDAC with AMD was analyzed using linear regression, while time-to-event analysis was used for T_R_. 32 AMD patients (mean ± SD; age:72 ± 6.3 years, VA:0.09 ± 0.08 logMAR) and 25 NV subjects (mean ± sd; age:65 ± 8.7 years, VA:0.049 ± 0.07 logMAR) were measured with the app. Controlling for age, VA, and cataract severity, the AMD presence was significantly associated with higher AUDAC (β = 0.41, 95% CI 0.18–0.64, *p* = 0.001) and with slower sensitivity recovery (β = 0.32, 95% CI 0.15–0.69, *p* = 0.004). DA measurements with the app were highly correlated with those obtained with AdaptDx—an established clinical device (n = 18, ρ = 0.87, *p* < 0.001). AMD classification accuracy using the app was 72%, which was comparable to the 71% accuracy of AdaptDx. Our findings indicate that the mobile app provided reliable and clinically meaningful DA measurements that were strongly correlated with the current standard of care in AMD.

## Introduction

Age-related Macular Degeneration (AMD) is one of the leading causes of blindness in older adults above 60 years of age. Late stage AMD affects about 2% of the US population, with the numbers projected to cross 3 million by 2030^1^. While there are no known cures for AMD at present, timely detection and monitoring can enable effective management of the condition to slow down its progression toward debilitating vision loss^2^. Visual acuity, the current standard of care, is not sensitive to changes in the retina due to AMD and thus is not a suitable visual function measure for screening or monitoring of AMD. Dark adaptation (DA) visual function on the other hand, being sensitive to changes in the retinas of AMD patients even during early stages, could be helpful for screening and monitoring of AMD progression.

Dark adaptation (DA) refers to the change in sensitivity of the visual system in darkness—the sensitivity increases over time until it reaches an absolute threshold value^3^. The sensitivity gain in the dark is initially guided by the response of cone photoreceptors, and then by the more sensitive rod photoreceptors^4,5^. Impairments or deficits in the DA visual function could be indicative of structural changes in the retina. For example, the DA response, specifically the rod-mediated DA is known to be impaired in AMD^6–10^. Furthermore, DA is likely to be affected in the early stages of the disease when other visual function measures such as visual acuity are usually not significantly affected^11,12^. Studies so far have shown that the abnormalities in the DA responses in older adults were seen before diagnosis of AMD^13^, were associated with known risk factors of AMD^14^, were correlated with AMD severity^8,15–18^, and could be observed longitudinally in AMD patients^19–22^. Thus, DA visual function can be important in diagnosis and monitoring of AMD and could serve as a more sensitive end point for clinical trials involving therapeutics for AMD patients^13,20^.

Psychophysical DA measurement process primarily involves: (i) exposing a region on the macula of the test eye to a bright light (bleaching), (ii) presenting a visual stimulus in the bleached area of the retina, eccentric to the fovea, and (iii) recording the recovery of the sensitivity over time in the dark. Various dedicated apparatus for DA measurement within lab and clinical settings have been developed broadly following these steps^23^. While effective in DA measurement^24,25^, clinical instruments for DA measurement are not currently used widely in clinics because of barriers due to high cost and accessibility, cumbersome and long measurement process, and requirement of trained and experienced technicians. Therefore the clinical instruments may not be suitable for mass screening or for home-based monitoring.

To address some of these concerns, we developed a method for DA measurement with a smartphone app and had previously performed its preliminary feasibility testing in people with normal vision without any retinal diseases^26^. Our previous work demonstrated that DA measurement was feasible with a smartphone and we could obtain repeatable measurements in people over a wide age range. Furthermore, we also introduced a novel way to quantify DA characteristics using the area under the DA curve (AUDAC), and validated it in standard of care instrument for DA measurement^27^. Using AUDAC, DA measurements with the mobile app could be readily quantified by a single number, such that larger AUDAC values indicate delayed or impaired DA response.

In this work, our goals were to perform clinical evaluation of the DA measurement mobile app and determine its efficacy in providing reliable and clinically meaningful DA measurements in AMD patients and older normally sighted adults without AMD. Given that the DA response is known to be impaired in AMD patients relative to normally sighted age-similar individuals without AMD, we hypothesized that app-based DA measurements will show significant impairment in the AMD patients relative to the older adults without AMD. We compared the app measurements with those obtained from a validated clinical instrument for DA, AdaptDx (Maculogix, Middletown, PA USA) with the expectations that the app measurements will be correlated with the measurements obtained from the current clinical standard device. We also investigated the effectiveness of the app DA measurements in classifying AMD presence in our study sample relative to the validated standard of care instrument.

## Methods

### Study design and participants

The data presented here were part of a larger study to evaluate the mobile app-based DA measurements. The study was approved by the Institutional Review Board of Mass Eye & Ear and followed the tenets of the Declaration of Helsinki. Written informed consent was obtained from all study participants. This was a cross-sectional study performed at the retina clinics of Mass Eye and Ear Infirmary, MA, USA. DA was measured in the selected eye of participants during a single visit to the retina clinic.

There were two groups: AMD patients and normally sighted older adults without AMD (NV). Individuals with current clinical diagnosis of AMD (at any stage) and visual acuity better than 20/40 were included. If both eyes met the inclusion criteria, the eye with better visual acuity (VA) was included as the study eye. If both eyes had the same VA, then one of the eyes was picked randomly as the study eye. Patients with other retinal disorders affecting vision (severe vision defects) such as inherited retinal disorders, diabetic retinopathy, glaucoma, refractive error > 6.0D or other disorders with visual field loss due to any cause were excluded. The NV group consisted of adults of age 50 or more without current clinical diagnosis of AMD or any of the other exclusion criterion mentioned above, and with VA better than 20/40 in the study eye.

The study participants were recruited from the patients visiting Mass Eye & Ear Infirmary who had consented to be approached for research studies. Study staff (AN, PS) prescreened the clinic and identified potential participants. They were approached by the study staff during the day of their visit in waiting room to ask for their willingness to go over the consent process. If they agreed, the study was explained in detail. If they agreed to participate, DA was measured on the same day during the visit. AMD was diagnosed and graded using color fundus photos and the AREDS 2 grading scale^28,29^ and details were previously described^30,31^.

### Measurement procedure

Details of the DA measurement procedure with the mobile app have been previously described^26,32^. Briefly, with the participant's fellow eye patched, the mobile device was placed at a distance of 40 cm from the test eye in a dark room. Bleaching was done by presenting a white screen with the highest brightness level (≈ 300 Cd/m^2^) for 2 min before starting the measurement. The app then presented a fixation cross and a test stimulus in the form of a flashing dot of 1.5° size at 5° from the fixation. The participant responded to a visible stimulus by tapping anywhere on the screen, and the current sensitivity level and the time elapsed from the start of the test was logged in the mobile device. The stimulus progressively got dimmer over time depending on the participant’s response. The measurement process continued until the sensitivity recovered by 3 log units or the measurement duration exceeded 20 min. The DA characteristics logged in the mobile device was exported to a desktop computer for further processing.

For a subset of participants, DA was also measured using the 20 min protocol of AdaptDx instrument during the same visit following the app-based measurement session. The test spot was 5° inferior to the fixation. The AdaptDx measurement session lasted until the time required for the sensitivity to recover by 3 log units (defined as the rod intercept time or RIT), with a maximum duration capped at 20 min. The instrument provided DA measurement output in terms of RIT, and if the sensitivity did not recover by 3 log units within 20 min, RIT was set at 20 min. DA measurement with the mobile device was done first and there was a gap of at least 30 min between the two measurement sessions.

DA measurements were conducted with dilated pupils. Dilating eye drops were administered after measurement of visual acuity. DA measurement was performed after a minimum of 30 min following dilation. In addition to the DA data, participant demographic information—age, gender, race, and vision measurements for the test eye—visual acuity, cataract grading including the presence of an intra-ocular lens (IOL) were acquired from the medical records. All subjects underwent a dilated eye exam on the same day of DA measurement and thus the same-day medical data was used in the analysis.

### Outcomes

We have 2 main outcome measures, one analogous to the conventional DA response time and one area under the curve measure, which was introduced and validated by us previously^27^. Photoreceptor (rod) sensitivity recovery is known to be the key parameter of a DA characteristics curve and conventionally, the time needed for this sensitivity to recover up to a certain threshold level in dark after bleaching is considered the outcome of the DA measurement^14,25^. The rod-intercept time (RIT), measured by the AdaptDx instrument, is the time required for the sensitivity to recover by 3 log units. Analogous to the conventional DA measure of RIT, we computed the overall response time (T_R_), which represented the time needed for the sensitivity to recover by 3 units when measuring with the mobile app. This was essentially the total measurement time for a given individual, capped at 20 min. Longer T_R_ values, similar to RIT, indicated delays (or impairments) in dark adaptation.

Additionally, the dark adaptation characteristics obtained from the mobile app were processed to compute the area under the DA curve (AUDAC). We have previously shown that AUDAC can be an effective way to quantify the DA response (not just in mobile device but with standard instrument as well), especially in AMD patients with slow recovery of rod sensitivity who do not cross the requisite sensitivity threshold within the maximum set measurement duration^27,32^. Theoretical range of AUDAC is from 0 to 1, with larger values indicating impaired DA response.

### Statistical analysis

We first performed linear regression to study the association between AUDAC with AMD presence. AMD presence was a binary variable that was formed by merging the stages of AMD: early, intermediate, and late stage to form one level (Yes), and the NV formed the other level (No). In the regression model, AUDAC (log transformed) was the dependent variable. Following predictors were examined for their association with AUDAC (log transformed) in univariate analysis: AMD presence (Y/N), age, gender (M/F), visual acuity (in logMAR), and cataract severity. Cataract grading was consolidated in 4 levels: none, mild (NS1), moderate (NS2 and above, including cortical cataracts), and with IOL. Since there were only a few cases of cortical cataracts, they were merged with moderate category together with eyes with NS2 grading. Significant predictors in univariate analysis were included in the final model. Model diagnostics were performed to ascertain the quality of the fit. AUDAC values were log transformed for ensuring a better model fit (normal residual distribution and homogeneity of variance).

Association of AMD presence (as the outcome variable) with AUDAC and other covariates such as age, visual acuity, and cataract levels was also investigated using a logistic regression model. Classification of AMD presence (Yes or No) based on the AUDAC values of app-based DA response was done using a logistic regression model in the framework of leave one out cross validation (LOOCV)^33^, and classification accuracy, sensitivity, and specificity were reported. AMD classification using on AUDAC for each sample was based on the outcome of the logistic regression model in LCOOV framework. This meant that classification was done by the computing odds for a sample in the data based on a model built using on all data except the given sample. If the computed odds were > 1, then it was classified as AMD, otherwise as NV. The AMD classification accuracy was compared to the no information rate—which was defined as the largest class proportion in the data (AMD vs. NV). Performance significantly better than no information rate would indicate that the classifier is doing better than just always picking a class that has the highest representation in the data^34^.

The overall measurement duration (T_R_), was a continuous variable. However, since the time of the measurement session was capped at 20 min, we treated T_R_ as a censored outcome measure and performed time-to-event analysis, where the event referred to the successful recovery of sensitivity by 3 log units within 20 min (test completion) and those who did not had their data censored. Therefore, the concept of “survival” in this analysis meant a lower likelihood of recovery of sensitivity over 3 log units within 20 min. Longer survival indicated slower or delayed DA. We performed Cox Proportional Hazard (CPH) regression to determine the association of AMD presence with survival, while controlling for other variable such as age, gender, visual acuity, and presence of IOL. Model diagnostics, including testing for proportional hazards assumption, were performed to ascertain the quality of the fit.

Correlation between mobile app and clinical device (AdaptDx) DA measurements were obtained using Spearman’s correlation coefficient (ρ) after adjusting for age (partial correlation between mobile app and AdaptDx measurements after controlling for the effect of age)^35^. Both, the conventional outcome measure of DA response in AdaptDx (RIT) and the area under the raw DA response curve measured by AdaptDx, were compared with the AUDAC values obtained from the mobile app measurements.

Regression results were reported in terms of the estimated coefficient, its 95% confidence interval (CI) and the corresponding P values. Two-sided P values less than 0.05 were considered statistically significant, and no adjustments were made for multiple analyses. Statistical analysis was conducted using statistical packages in R version 4.0.4 (The R Foundation).

### Data sharing statement

The data generated during and analyzed during the current study are not publicly available due to institutional restrictions because of the data containing information that could compromise research participant privacy/consent, but part of it (de-identified) could be made available from the corresponding author on reasonable request.

## Results

DA measurement with the mobile device was performed in 59 individuals (25 NV and 34 AMD patients). Of these, data for 2 AMD patients were excluded because of incomplete measurement (measurement manually terminated before the termination criteria were met). Thus, data for 57 individuals, 32 AMD (1 Early, 29 Intermediate, and 2 Late) and 25 NV, were available for analysis (Table 1). The mean age was significantly larger in the AMD group compared to the NV group (mean ± SD; AMD: 72 ± 6 years, NV: 65 ± 9 years, t = − 3.28, df = 42.2, *p* = 0.002). The proportion of females was higher in the AMD group (75%) compared to the NV group (48%), but the difference was not statistically significant. The vast majority of the sample were Caucasians (NV: 84%, AMD: 91%). There was a slight but statistically significant difference in the median VA between the two subject groups (NV: 0.0 logMAR, AMD: 0.1 logMAR, W = 272, *p* = 0.036), even though only people with VA better than 20/40 (or 0.3 logMAR) were included in the study in both groups. Study eye for 1 participant was graded at a level higher than NS2+ for cataract severity, and 2 had cortical cataracts. About 24% of NV and 28% of AMD patients had an IOL in the test eye, but the proportions of individuals with an IOL were not significantly different between the two subject groups. However, individuals with IOL were significantly older (average age 75 years) than other 3 cataract severity levels (df = 3, F = 6.9, *p* < 0.001).

**Table 1:** Study sample characteristics.

	NV	AMD
N	25	32 (1 early, 29 intermediate, 2 late)
Females (%)	12 (48%)	24 (75%)
Race/ethnicity	Asian: 2	Asian: 1
	Black: 0	Black: 1
	Caucasian: 21	Caucasian: 29
	Latino: 1	Latino: 0
	NA: 1	NA: 1
Age (years)	Mean: 65	Mean: 72
	SD: 8.7	SD: 6.3
	Range: 50–84	Range: 56–85
VA (logMAR)	Median: 0.0	Median: 0.1
	IQR: 0.1	IQR: 0.13
	Range: − 0.06 to 0.18	Range: − 0.04 to 0.22
Cataract status	None: 6	None: 2
	Mild: 6	Mild: 13
	Moderate:7	Moderate: 8
	IOL: 6	IOL: 9

The sample mean (SD) [min, max] AUDAC for AMD and NV groups were 0.35 (0.14) [0.09, 0.39] and 0.20 (0.07) [0.13, 0.59], respectively. In a univariate analysis, age, AMD presence, VA, and IOL presence were the significant predictors of elevated AUDAC. Particularly, AUDAC increased by 0.08 with a decade advance in age, by 0.06 per 0.01 increase in logMAR VA, and by 0.15 in those who had IOL relative to those with no cataract. In a multivariate analysis, controlling for the age, VA, and cataract severity factor (includes IOL presence), the presence of AMD was significantly associated with higher AUDAC (β = 0.41, 95% CI 0.18–0.64, *p* = 0.001). The estimated marginal mean AUDAC (95% CI) for AMD and NV was 0.31 (0.27–0.36) and 0.21 (0.17–0.24), respectively. Thus, the average AUDAC in the AMD patients was significantly higher, by about 50% compared to NV subjects, after controlling for age, VA, and cataract grading. Subject age, visual acuity, and cataract severity were not significant predictors of AUDAC in the multivariate regression model (details of the regression model for AUDAC are shown in Table 2).

**Table 2:** Regression model coefficients for AUDAC (log transformed).

	Est	95% CI	*P* value
Age (decade)	0.12	− 0.04 to 0.28	0.143
AMD presence	0.41	0.18 to 0.64	0.001
VA (logMAR)	0.99	− 0.42 to 2.41	0.163
Cataract level—mild	− 0.05	− 0.39 to 0.29	0.757
Cataract level—moderate	0.10	− 0.24 to 0.45	0.556
IOL presence	0.14	− 0.24 to 0.53	0.456

In a univariate logistic regression model, the odds of AMD presence significantly increased with a unit increase in the log AUDAC value (Odds Ratio = 26.3, 95% CI 5.4–198, *p* < 0.001). This meant that the odds ratio associated with 1% increase in the original (untransformed) AUDAC value was 1.033 (can be computed as e^(log(23.3)×log(1.01))^). When controlling for age, visual acuity, and cataract levels, the odds ratio for AUDAC (log transformed) was still statistically significant (Odds Ratio = 25.1, 95% CI: 3.7–292, *p* = 0.003). This amounted to a very similar odds ratio of 1.032 for 1% increase in the original untransformed AUDAC value. None of the other covariates were found to be statistically significant. Using AUDAC (log transformed) in a LOOCV framework to classify AMD presence resulted in a sensitivity of 75% (24 correctly classified out of 32 AMD), specificity of 68% (17 correctly classified out of 25 NV), and an overall accuracy of 72% (41 out of 57). The overall classification accuracy of 72% (95% CI 58–83%) was significantly better than the no-information rate of 56% (*p* = 0.01). Accounting for cataract levels (including IOL presence) in the prediction model resulted in a sensitivity of 78%, specificity of 72%, and an overall accuracy of 75% in classifying AMD presence using AUDAC of mobile app DA measurements.

Sample mean (SD) T_R_ for AMD and NV groups was 16.5 (4.0) minutes and 11.9 (3.4) minutes, respectively. However, the mean values were affected by the measurement duration cutoff limit of 20 min, as 17 out of 32 AMD patients (53%) and 1 out of 25 NV subjects (4%) failed to finish before 20 min – a significant difference (χ^2^ = 13.5, df = 1, *p* < 0.001). Univariate analysis was performed to test the association of T_R_ with following predictors of interest: age, gender, visual acuity, AMD presence, and cataract levels including IOL presence. Of these, age, visual acuity, AMD presence and IOL presence were determined to be significant predictors of T_R_. Controlling for age, cataract levels (including IOL presence), and visual acuity in a CPH regression model, AMD presence was significantly associated with lower probability of sensitivity recovery within 20 min (β = 0.32, 95% CI 0.15–0.69, *p* = 0.004) (Table 3).

**Table 3:** Cox Proportional Hazard Regression for T_R_.

	Estimate exp(coeff.)	95% CI	*P* value
Age (years)	0.97	0.93–1.01	0.119
AMD presence	0.32	0.15–0.69	0.004
VA (logMAR)	0.14	0.00–27.0	0.468
Cataract level: mild	1.30	0.49–3.43	0.601
Cataract level: moderate	1.01	0.37–2.76	0.989
Presence of IOL	0.68	0.21–2.19	0.519

**Figure 1:**
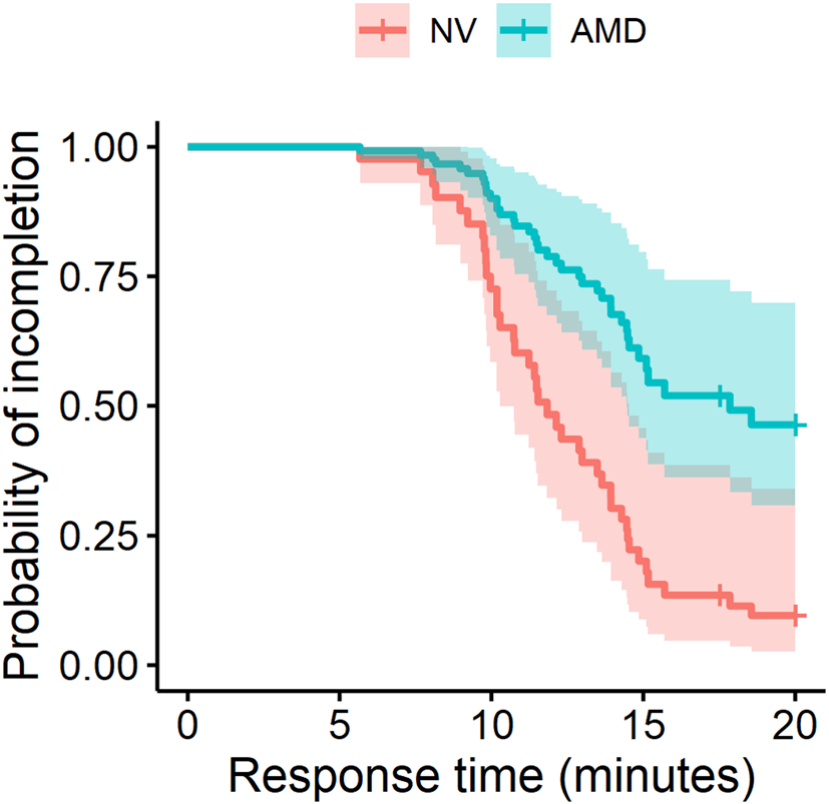
Age-adjusted survival curves, indicating the probability of reaching the 3 log unit threshold sensitivity for a range of time durations. The survival curves were significantly different, which indicated delays in the DA for AMD patients compared to the NV subjects.

Survival curves adjusted for age and visual acuity show that the probability of failing to reach 3 log unit threshold was higher in AMD patients than NV subjects (Fig. 1). The survival probability, or the probability that the sensitivity had not recovered by 3 log units at 20 min time point was 0.46 and 0.1 for AMD patients and NV subjects, respectively. At 15 min, this was 0.57 and 0.18 respectively for AMD and NV subjects. The two curves first start to diverge at around 5 min time point and after 15 min, remain approximately parallel to each other.

**Figure 2:**
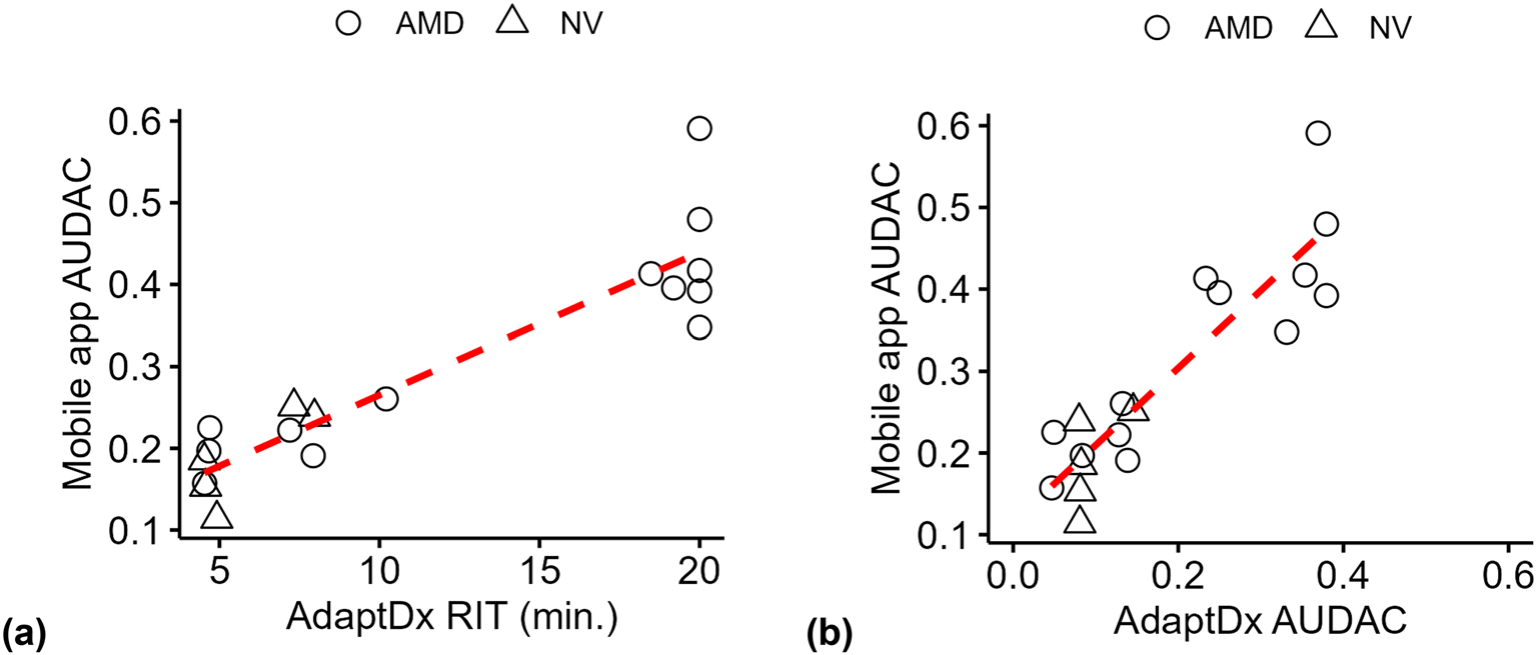
Age-adjusted correlation between mobile app and AdaptDx measurements (n = 18). AUDAC of the mobile app measurements were highly correlated with same day AdaptDx RIT values (**a**), and with the AUDAC values computed from the AdaptDx raw DA characteristics (**b**).

DA was measured with AdaptDx instrument for a total of 55 eyes (both eyes for 26 individuals and one eye for 9 individuals), of which 29 eyes had a diagnosis of AMD and 26 were NV eyes. Same day AdaptDx and mobile app measurements were available for 18 individual eyes (13 AMD, 5 Controls). There was a strong correlation between app measurements (AUDAC) and the AdaptDx RIT (ρ = 0.87, S = 6.7, *p* < 0.001) (Fig. 2a). Out of 13 AMD patients, 5 recorded an RIT value of 20 min. After converting the raw DA characteristics obtained from the AdaptDx instrument to AUDAC values, they were still significantly correlated with the AUDAC values of the mobile app measurements (ρ = 0.78, S = 4.9, *p* < 0.001) (Fig. 2b).

Using the predefined criterion of RIT > 6.5 min to classify AMD presence in all eyes measured via AdaptDx instrument^25^ resulted in a sensitivity of 69% (20 correctly classified out of 29 AMD), specificity of 73% (19 correctly classified out of 26 Controls), and an overall accuracy of 71% (39 out of 55). The classification accuracy of 71% (95% CI 57–82%) was significantly better than the no-information rate of 53% (*p* = 0.004). Comparison of AMD presence detection performance based on mobile app AUDAC and AdaptDx clinical instrument RIT values is summarized in Table 4.

**Table 4:** Comparison of AMD presence detection performance.

	Mobile app AUDAC	Clinical DA instrument RIT
N^a^ (eyes)	57 (AMD: 32, NV:25)	55 (AMD: 29, NV: 26)
Sensitivity	75%	69%
Specificity	68%	73%
Accuracy	72%	71%
No information rate	56%	53%

^a^Data reported for different set of eyes for two methods with an overlap of 18 eyes.

## Discussion

This study evaluated the mobile app for DA measurement in older adults with normal visual acuity, with and without AMD, and found that there were significant differences in the DA response as measured by the app between the two groups. The DA response indicated delayed dark adaptation in AMD patients than the NV group, after controlling for other factors and covariates. This finding is consistent with the large amount of evidence showing impaired DA in AMD patients^6–10^. Moreover, the app measurements were highly consistent with same-day measurements from a standard of care clinical instrument for DA measurement. Together, these findings indicate that the app measurements are valid and clinically meaningful.

Various physical and physiological factors affect dark adaptation^32,36,37^. Age is one of the prominent factors affecting DA, with studies reporting slower response and/or elevated rod and cone thresholds with increasing age^38–40^. Consistent with the previous reports, we also observed that the mobile app AUDAC values increased with subject age, indicating that the DA response got worse with advanced age. In our sample, AMD patients were older than the NV subjects, so the age was a confounder. Also, the visual acuity of AMD patients in our sample was slightly but statistically significantly worse than the NV subjects, despite all the participants having normal visual acuity. Cataract severity is also know to affect the DA response, and particularly, the presence of IOL delayed the DA response^41^. Typically, older individuals had higher cataract severity or had IOL in the study eyes. Therefore, it was important to control for age, VA, and cataract levels (including IOL presence) when interpreting DA measurements. In our case, the effect of AMD presence on the DA measurements was significant even after controlling for these key factors and covariates, which indicates the robustness of the DA measurement obtained via mobile app.

AdaptDx is a validated clinical device for DA measurement and high degree of correlation between the mobile app measurements with same-day measurements from AdaptDx, as seen in Fig. 2, further demonstrate the validity of DA measurements with the mobile app. It should be noted that due to differences in the measurement protocols between the two methods (for example differences in bleaching levels, stimulus range, measurement location, and the DA outcome measure), a direct numerical comparison is not meaningful. In fact, different protocols on the same device lead to different results^37^. The correlation analysis presented above (Spearman’s ρ) indicates a monotonic relationship between mobile app and AdaptDx measurements, which means that for the given eye, both devices tend to assess relative impairments in the DA at the same level. So an eye with impaired DA response as measured with the mobile app also tends to have relatively impaired DA response as measured by AdaptDx. The significant and strong correlation between the two devices in Fig. 2 shows agreement between the two methods regarding relative DA impairments in the eyes that were measured.

In our study sample, the sensitivity and the specificity of AMD detection using the mobile app measurements were comparable to those obtained with the AdaptDx measurements. The sensitivity of AdaptDx observed in our data was lower than the values reported in the previous studies of AMD patients. Based on the same RIT > 6.5 min criterion using the AdaptDx instrument, Jackson et al. reported 91% sensitivity for AMD detection in 127 AMD patients and 21 normal adults^25^. However, the VA inclusion cutoff for AMD patients was 20/100 in the study by Jackson et al., which was more relaxed than our inclusion limit (VA better than 20/40). Therefore it is possible that their study involved some late AMD patients, where AMD could be more easily detected due to larger impairments in the DA. Similar to that study, our previous study using AdaptDx with the same RIT criterion of 6.5 min for a different set of 98 eyes with AMD and 38 NV controls showed 77% sensitivity and 82% specificity^27^. It should be noted that unlike the sensitivity and specificity values using AdaptDx found in this study, the app AUDAC values were not categorized using a pre-defined cut-point, but were classified based on the fitted logistic regression models involving log of app AUDAC values. For each data point, the logistic regression model was fitted with the rest of the data and prediction was made for comparison with the said data point.

Additionally, in this study, we show that survival analysis could be used in the context of DA measurements involving censored data due to the limits on the measurement duration. One could argue that increasing the measurement duration could increase the likelihood of successfully measuring the sensitivity cross the 3 log unit threshold. However, further increasing the measurement duration makes an already tedious DA measurement process highly impractical in clinical studies. Therefore the goal here was to try to reduce the testing burden by making the process easier and more efficient. The tradeoff of lower overall measurement duration is the increase in probability of censoring the data, for which survival analysis is suitable. In addition to dealing with censored data, survival analysis could also potentially indicate areas of for further improving the efficiency. Since the survival curves for the two subject groups were almost flat beyond the 15 min mark (Fig. 1), the overall measurement time with the app could be reduced to around 15 min, down from 20 min, without losing substantial information.

Our previous work on the development of the mobile app showed the feasibility of measuring DA response on conventional smartphones and demonstrated the repeatability of the app in measuring DA^26^. This current work is the next step towards clinical translation, which showed that clinically meaningful results could be obtained with the mobile app in AMD patients, similar to those obtained with the standard of care clinical instrument. Despite its promise as a valuable functional biomarker in diagnosis and prognosis of AMD, currently, DA measurement is not typically included as part of routine workflow in ophthalmology clinics because testing requires trained administrator and tends to be tedious. The mobile app could potentially addresses the unmet needs of measuring DA in clinical settings, by simplifying the measurement process and making it more accessible. This study was conducted in patients during their routine clinical visit, by incorporating mobile app-based DA measurement during the regular workflow of the clinic. The findings demonstrate that the mobile app could potentially facilitate measurement of DA in clinics, thereby providing a valuable piece of information in detecting and/or managing AMD.

There are some limitations of this study. First, the sample size for comparing the app measurements with clinical instrument was small, with a heavy representation of intermediate AMD patients. This was possibly because intermediate AMD formed the bulk of the AMD patients referred to the retina clinic who qualified for this study. Due to our strict inclusion criterion regarding the VA in the study eye, we ended up excluding most late AMD patients. Since one of the goals of the mobile app evaluation was to determine its diagnostic ability, the inclusion criterion was set for participants to have normal visual acuity (better than 20/40), no scotomas, and no other vision defects. Thus, we also assumed that the fixation stability was normal is our study subjects and the measurement setup did not include eye movement monitoring. Implementation of eye movement monitoring during the test could further enhance reliability of the measurements. Another limitation of this study was that we only observed the cross-sectional DA response, without correlating it to the structural changes/features in the retinas of the participants. Future work involves addressing these limitations.
